# Sequential Use of Carfilzomib and Pomalidomide in Relapsed Multiple Myeloma: A Report from the Canadian Myeloma Research Group (CMRG) Database

**DOI:** 10.3390/curroncol29030132

**Published:** 2022-03-02

**Authors:** Arleigh McCurdy, Christopher P. Venner, Esther Masih-Khan, Martha Louzada, Richard LeBlanc, Michael Sebag, Kevin Song, Victor H. Jimenez-Zepeda, Rami Kotb, Moustafa Kardjadj, Hira Mian, Darrell White, Julie Stakiw, Muhammad Aslam, Anthony Reiman, Engin Gul, Donna Reece

**Affiliations:** 1 Department of Medicine, The Ottawa Hospital, Ottawa, ON K1N 6N5, Canada; amccurdy@toh.ca; 2 Cross Cancer Institute, University of Alberta, Edmonton, AB T6G 1Z2, Canada; christopher.venner@bccancer.bc.ca; 3 Princess Margaret Cancer Centre, Toronto, ON M5G 2C1, Canada; donna.reece@uhn.ca; 4 Canadian Myeloma Research Group, Vaughn, ON L4K 4L8, Canada; biostats@cmrg.ca (M.K.); egul@cmrg.ca (E.G.); 5 London Regional Cancer Center, London, ON N6A 5W9, Canada; martha.louzada@lhsc.on.ca; 6 Maisonneuve-Rosemont Hospital Research Centre, University of Montreal, Montreal, QC H1T 2M4, Canada; richard.leblanc.med2@ssss.gouv.qc.ca; 7 Department of Medicine, McGill University, Montreal, QC H3A 0G4, Canada; michael.sebag@mcgill.ca; 8 BC Cancer Agency, Vancouver General Hospital, Vancouver, BC V5Z 4E6, Canada; ksong@bccancer.bc.ca; 9 Arnie Charbonneau Cancer Institute, University of Calgary, Calgary, AB T2N 4Z6, Canada; victor.zepeda@albertahealthservices.ca; 10 Cancer Care Manitoba, Winnipeg, MB R3E 0V9, Canada; rkotb@cancercare.mb.ca; 11 Juravinski Cancer Center, Hamilton, ON L8V 5C2, Canada; hira.mian@medportal.ca; 12 Queen Elizabeth II Health Sciences Centre, Dalhousie University, Halifax, NS B3H 1V7, Canada; darrell.white@nshealth.ca; 13 Saskatoon Cancer Centre, University of Saskatchewan, Saskatoon, SK S7N 4H4, Canada; julie.stakiw@saskcancer.ca; 14 Allan Blair Cancer Centre, Regina, SK S4T 7T1, Canada; muhammad.aslam@saskcancer.ca; 15 Department of Oncology, Saint John Regional Hospital, Saint John, NB E2L 4L2, Canada; anthony.reiman@horizonnb.ca

**Keywords:** multiple myeloma, relapsed/refractory, pomalidomide, carfilzomib, sequence

## Abstract

The treatment of multiple myeloma has dramatically improved due to the availability of novel therapies that are highly effective and are quickly moving into first-line therapy. The Canadian Agency for Drugs and Technologies in Health (CADTH) recently recommended that patients who receive daratumumab should only be eligible to receive either carfilzomib or pomalidomide but not both, for relapsed MM. In order to assess the efficacy of these two agents in the relapsed setting, we utilized our national myeloma database. A total of 121 patients were reviewed, 49 patients received CAR- before POM-based (CAR-POM), and 73 patients received POM- before CAR-based (POM-CAR) therapy. In the groups selected, the median PFS was 4.93 months (95% CI, 2.76–7.07) and 5.36 months (95% CI, 3.75–6.94) for CAR-POM and POM-CAR, respectively. The median OS for patients treated with CAR-POM was 11.01 months (95% CI, 4.50–19.13), and for patients treated with POM-CAR the median OS was 10.98 months (95% CI, 8.98–19.17). In this real-world observational study, we demonstrated that both CAR- and POM-based therapies, irrespective of the order in which they were used, were effective treatment options for patients with advanced relapsed MM.

## 1. Introduction

Multiple myeloma (MM) is a plasma cell neoplasm characterized by a clonal proliferation of plasma cells in the bone marrow. Despite significant advances in treatment over the last decade, MM unfortunately remains incurable [1]. For most patients, the course of the illness in MM is one of treatment with chemotherapy followed by a period of remission, and subsequently, disease relapse requiring additional therapy. Throughout the disease course, the periods of remission become shorter as MM becomes refractory to treatment [2]. The novel agents, including proteasome inhibitors (PIs), immunomodulatory agents (IMIDs), and most recently, monoclonal antibodies (MABs), have revolutionized MM treatment and resulted in unprecedented improvements in survival [3].

In the Canadian landscape, bortezomib, lenalidomide, and daratumumab (DARA) are used as treatment at diagnosis and at first relapse, in various combinations based on efficacy shown in large, randomized trials [4,5,6,7]. However, treatment for second and third relapses and beyond is less standardized, due to both the heterogeneity of the illness at that point in a patient’s clinical course and a data gap in optimal sequencing of the available agents approved to treat MM in this setting. 

The second-generation PI carfilzomib (CAR) and third-generation IMiD pomalidomide (POM) are approved by Health Canada and are typically the most used agents in this space based on the data of their respective efficacies in relapsed myeloma. However, the ideal order with which these agents are used and their efficacy when used in sequence with each other is unknown. As DARA-based regimens may be effective for a prolonged period, the Canadian Agency for Drugs and Technologies in Health (CADTH), which provides reimbursement guidelines to the provincial ministries of health, recommended, in 2019, against open sequencing of drugs in relapsed MM. Specifically, patients who receive DARA would be eligible only for publicly reimbursed CAR or POM, but not both, for relapsed MM. 

Given the known heterogeneity of MM and the uncertainty regarding the impact of this new restriction on patient outcomes, we utilized the national Canadian Myeloma Research Group database (CMRG-DB) to assess individual patients who received both of these agents as treatment for RRMM. The goal of our study was to evaluate the efficacy of these two commonly used treatments in the relapsed setting: (1) CAR-based before POM-based therapy and (2) POM-based before CAR-based therapy.

## 2. Materials and Methods

### 2.1. CMRG Database 

This study analysed patients included in the CMRG Database, a Canadian web-based repository of retrospective and prospective data on over 7000 patients with MM from 14 academic centres. Data was collected on patient demographics, type and duration of treatments received, response to therapy according to IMWG criteria, duration of response, and survival/death. All patient data reported to the CMRG Database by participating centers have obtained informed consent for use of their information for research purposes. The approval for this review was from the Ottawa Health Science Network Research Ethics Board (OHSN-REB) as per the approved governance structure of the CMRG Database.

### 2.2. Patients

The study cohort consisted of adults (≥18 years of age) with relapsed MM in the CMRG Database who had received both CAR-based and POM-based therapy, regardless of which therapy came first or at which line of treatment. CAR or POM given in combination with unapproved clinical trial drugs were excluded. The therapies were both given during the period between March 2012 to June 2020 and there was no minimum duration for the first or subsequent therapy. Disease progression was defined as per IMWG consensus guidelines. Fluorescence in situ hybridization (FISH) was performed at diagnosis and translocation t(4:14) or t(14:16), or deletion 17p were classified as having high-risk disease. All other cases were considered as standard risk, according to IMWG guidelines.

### 2.3. Endpoints and Statistical Analysis

The primary endpoint of this study was to assess the overall response rate (ORR) in two groups of patients with RRMM who received both CAR and POM for RRMM. One group received CAR after POM, and one group received POM after CAR. Secondary endpoints assessed in the same groups were progression free survival (PFS) and overall survival (OS). ORR was defined as an equal or greater than partial response (PR). PFS was defined as the time from the first dose of therapy given until disease progression or death from any cause. PFS2 was defined as the time from initiation of first therapy to progression on subsequent therapy. OS was the time from the start of first therapy until death from any cause or censored at date of last follow-up.

Statistical analyses were performed using R core team 2020 (R-4.1.1), Vienna, Austria and RStudio team 2019 (RStudio-1.4.1717), Boston, MA, USA for Windows. All tests were 2-sided, *p* < 0.05 were considered to indicate a statistically significant result. Categorical comparisons were performed using the chi-square test and continuous ones using ANOVA. PFS and OS rates were calculated using the Kaplan–Meier product-limit method and the log-rank statistic was used for the comparison of PFS and OS curves. 

## 3. Results

A total of 121 patients were included in this analysis, 49 patients were treated with CAR-based followed by POM-based therapies, and 72 patients were treated with POM-based followed by CAR-based therapies. The baseline characteristics and detail of therapies for the two groups are summarized in Table 1. The median age for the cohort at diagnosis was 60 years (34–82 years) and 58.7% of the patients were males. In the CAR-POM group, CAR was given as a median third-line treatment and POM as a fourth-line treatment. In the POM-CAR group, POM was given as a median fourth-line treatment and CAR was given as a median fifth-line treatment. In 78 of 121 patients (65%), the two therapies were directly sequential, 40 patient (81.6%) for the CAR-POM group, and 38 patients (52.8%) in the POM-CAR group. In the CAR-POM group, 41 patients (83.7%) were lenalidomide exposed, 33 patients (67.3%) were refractory to lenalidomide, and 5 patients (10.2%) were daratumumab exposed. In the POM-CAR group also, a high number of patients were lenalidomide exposed (*n* = 70, 97.2%) and refractory (*n* = 63, 87.5%). 

The ORR was similar for both CAR- and POM-based therapies in the two groups (Table 2). A partial or greater than partial response for CAR was seen in 62.0% patients in the CAR-POM group and in 47.1% patients in the POM-CAR group. Similarly, ≥PR response for POM-based therapies was seen in 48.9% of patients in the CAR-POM group and 50.0% of patients in the POM-CAR group. However, when deeper responses were assessed, equal or greater than very good partial responses (≥VGPR) were higher for the therapy that was received first (Table 2). 

**Figure 1:**
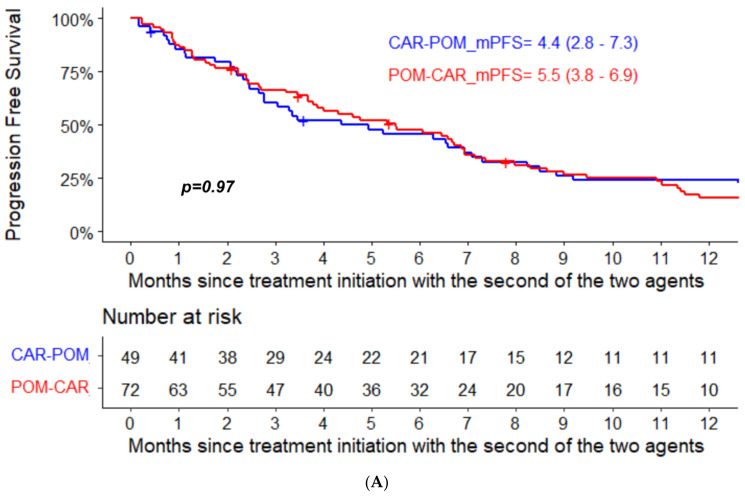

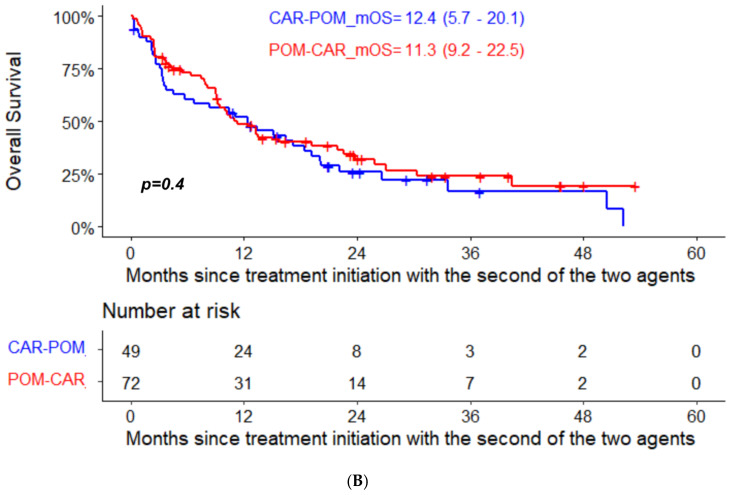
(**A**) PFS and (**B**) OS analysis from treatment initiation with the second of the two agents in the two groups.

**Figure 2:**
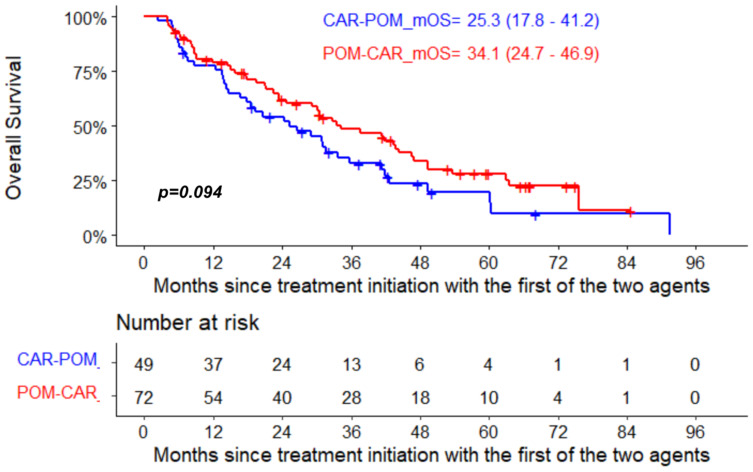
Landmark OS analysis from treatment initiation with the first of the two agents in the two groups.

The median PFS for pomalidomide therapy in the POM-CAR group was 6.4 months (95% CI, 4.0–12.8) and 4.4 months in the CAR-POM group (95% CI, 2.8–7.3); the median PFS for CAR therapy was 7.4 months (95% CI, 5.9–11.3) in the CAR-POM group and 5.5 months (95% CI, 3.8–6.9) in the POM-CAR group (Figure 1A). In a subset analysis for PFS2 in patients that received both drugs directly sequential to each other, CAR-POM (*n* = 40) and POM-CAR (*n* = 38) gave a PFS2 of 13.2 months and 13.0 months that was not significantly different (Table 1). The median OS for patients treated with POM after CAR was 11.3 months (95% CI, 9.2–22.5), and for patients treated with CAR after POM the median OS was 12.4 months (95% CI, 5.7–20.1) (Figure 1B). In a landmark analysis using the date of the treatment initiation with the first of the two agents to death or last follow-up, the median OS of patients treated with CAR after POM was 34.1 months (95% CI 24.7–46.9) and 25.3 months (95% CI 17.8–41.2) for patients treated with POM after CAR (*p* = 0.094) (Figure 2).

## 4. Discussion

In this real-world observational study, we demonstrated that both CAR- and POM-based therapies were effective treatment options for patients with advanced relapsed MM. Each therapy produced responses in approximately 50% of patients with a median PFS of about 5 months and median OS of 11 months. These patients were heavily pretreated, with a median of four and five prior treatment lines in the CAR-POM and POM-CAR groups, respectively, with the majority of patients in both cohorts being exposed and refractory to lenalidomide. In addition, a landmark analysis showed that using both agents sequentially late in the disease course provided reasonable OS outcomes, regardless of the order in which they are used. As such, limiting patients to a choice of either CAR or POM, but not both, as potential therapeutic options for RRMM could result in meaningful impacts on patients, some of whom have prolonged responses to these agents. 

These results are comparable to those noted in prospective clinical trials leading to the approval and reimbursement of these agents in this setting [8,9]. The MM-003 Phase III trial showed a median PFS of 3.8 months vs. 1.9 months (and a median OS of 11.9 months (10.4–15.5) vs. 7.8 months for PomDex and high-dose dexamethasone, respectively) [9]. The Carfilzomib arm of the ENDEAVOR Phase III trial included 117 patients (24%) who were refractory to Lenalidomide, resulting in a median PFS of 8.6 months [10] and a median OS of 29.2 [11] months; however, this study was limited to patients receiving from one to three prior lines. Perhaps more reflective of the patient population in our study, the phase III FOCUS trial randomized patients with a median of five prior lines to Carfilzomib or low dose corticosteroids +/− cyclophosphamide, and the median PFS of the Carfilzomib group was 3.7 months with median OS 10.2 months [12]. 

With the advent of innovative novel therapeutics in MM treatment including novel cellular therapies and immunotherapeutic platforms, the cost of MM therapy continues to rise and, in publicly reimbursed health care systems such ours, we recognize that ongoing, robust, evidence-based evaluation of the efficacy and value of these expensive medications is required. The use of real-world data can help determine the impact of funding decisions on the outcome of patients treated in a publicly funded health care system. 

## 5. Conclusions

In summary, our results demonstrate that both pomalidomide- and carfilzomib-based regimens have efficacy comparable to that seen in clinical trials when used in a real-world setting in patients with heavily treated refractory myeloma, regardless of the order in which they are used. Strengths of our study include robustly collected disease, treatment, and response specific data. Our study has several important limitations including its retrospective nature, small size, and patients previously treated with daratumumab-containing regimens, which were not present in our database in significant numbers due to a lack of reimbursement and availability during the study period. That said, such patients were also not reflected in the populations included in the clinical trials that led to current approvals and funding. Additional studies with longer follow-up are required to assess the optimal use of these two agents.

## Figures and Tables

**Table 1:** Baseline characteristics and treatment details of all patients stratified by the sequential use of carfilzomib and pomalidomide based therapies.

Baseline Characteristics	All	CAR-POM	POM-CAR
	(*n* = 121)	(*n* = 49)	(*n* = 72)
**Age at Diagnosis, median (range)**	60 (34–82)	63 (41–80)	56 (34–82)
**Age at Initiation of first therapy CAR/POM, median (range)**	65 (39–84)	67 (48–82)	64 (39–84)
**Male, N (%)**	71 (58.7)	26 (53.1)	45 (62.5)
**MM Subtype, N (%)**			
**IgG**	68 (59.1)	28 (60.9)	40 (58.0)
**IgA**	30 (26.1)	13 (28.3)	17 (24.6)
**IgD**	1 (0.9)	0 (0)	1 (1.5)
**FLC**	16 (13.9)	5 (10.9)	11 (15.9)
**Unknown**	6	3	3
**ISS Stage, N (%)**			
**I**	27 (29.6)	8 (19.5)	19 (34.6)
**II**	35 (39.0)	16 (39.0)	19 (34.6)
**III**	34 (31.5)	17 (41.5)	17 (30.9)
**Unknown**	25	8	17
**Previous Treatment, N (%)**			
**Lenalidomide exposed**	111 (91.7)	41 (83.7)	70 (97.2)
**Lenalidomide refractory**	96 (79.3)	33 (67.3)	73 (87.5)
**Daratumumab exposed**	8 (6.6)	5 (10.2)	3 (4.2)
Cytogenetics *, N (%)			
**High risk**	24 (25.5)	15 (42.9)	9 (15.3)
**Standard risk**	70 (74.5)	20 (57.1)	50 (84.8)
**Unknown**	27	14	13
**POM-based Regimen**			
**PomC**	2 (1.7)	1 (2.0)	1 (1.4)
**PomCD/P**	62 (51.2)	36 (73.5)	26 (36.1)
**PomD**	53 (43.8)	11 (22.5)	42 (58.3)
**PomVD**	4 (3.3)	1 (2.0)	3 (4.2)
**CAR-based Regimen**			
**K**	7 (5.8)	1 (2.0)	6 (8.3)
**KCD**	20 (16.5)	12 (24.5)	8 (11.1)
**KD/P**	76 (62.8)	22 (44.9)	54 (75.0)
**KRD**	18(14.9)	14 (28.6)	4 (5.6)
**Line of First Treatment, median (range)**	3 (2–9)	3 (2–6)	4 (2–9)
**Subsequent Treatments, N (%)**			
**Lenalidomide**	8 (6.6)	4 (8.2)	4 (5.6)
**Pomalidomide**	71 (58.7)	49 (100)	22 (30.6)
**Bortezomib**	20 (16.5)	6 (12.2)	14 (19.4)
**Carfilzomib**	76 (62.8)	4 (8.2)	72 (100)
**Ixazomib**	12 (9.9)	3 (6.1)	9 (12.5)
**Anti-CD38**	43 (35.5)	16 (32.7)	27 (37.5)
**Anti-BCMA**	9 (7.4)	3 (6.1)	6 (8.3)

* High risk cytogenetics defined as del 17p, t (4;14) and/or t (14;16). Abbreviations: MM, multiple myeloma; ISS, international staging system P, pomalidomide; C, cyclophosphamide; D, dexamethasone; P, prednisone; V, bortezomib; K, carfilzomib; R, lenalidomide.

**Table 2:** Therapy responses of carfilzomib- and pomalidomide-based therapies in the CAR-POM and POM-CAR groups.

Therapy Response	All (*n* = 121)	CAR-POM (*n* = 49)	POM-CAR (*n* = 72)
**Carfilzomib-based**			
ORR (≥PR), *n* (%) *	62 (52.1)	29 (62.0)	33 (47.1)
**≥VGPR, *n* (%)**	25 (21.0) ^α^	18 (38.3) ^β^	7 (10.0) ^β^
**Median PFS (95% CI)**	6.4 (5.1–7.4)	7.4 (5.9–11.3)	5.5 (3.8–6.9)
**Pomalidomide-based**			
**ORR (≥PR), *n* (%)**	57 (49.6)	22 (48.9)	35 (50.0)
**≥VGPR, *n* (%)**	19 (16.5) ^α^	5 (11.1) ^γ^	14 (20.3) ^γ^
**Median PFS (95% CI)**	5.7 (4.0–8.2)	4.4 (2.8–7.3)	6.4 (4.0–12.8)
**	**All** **(*n* = 78)**	**CAR-POM** **(*n* = 40)**	**POM-CAR** **(*n* = 38)**
Median PFS 2 (95% CI) **	13.0 (10.4–24.2)	13.2 (10.4–24.2)	13.0 (8.2–31.1)

* Percentage based on evaluable patients. ** Subset analysis of patients that received direct sequential treatment. ^α^ No significant difference (chi-square test, *p* = 0.31) ^γ^ No significant difference (chi-square test, *p* = 0.17) ^β^ Significant difference (chi-square test, *p* = 0.003).

## Data Availability

Canadian Myeloma Research Group database (CMRG-DB) governance and research ethics boards at data contributing sites, do not allow patient level data to be shared. Any aggregate data supporting the findings of this study can be available from the corresponding author upon reasonable request.
